# pH-induced conformational changes in the selectivity filter of a potassium channel lead to alterations in its selectivity and permeation properties

**DOI:** 10.3389/fphar.2024.1499383

**Published:** 2025-01-06

**Authors:** Carlos Coll-Díez, Ana Marcela Giudici, Alberto Potenza, José Manuel González-Ros, José Antonio Poveda

**Affiliations:** IDiBE—Instituto de Investigación, Desarrollo e Innovación en Biotecnología Sanitaria de Elche, Universidad Miguel Hernández, Elche, Spain

**Keywords:** conformational plasticity, ion selectivity, ion conduction, induced-fit conformation, ion binding, thermal denaturation assay

## Abstract

The Selectivity Filter (SF) in tetrameric K^+^ channels, has a highly conserved sequence, TVGYG, at the extracellular entry to the channel pore region. There, the backbone carbonyl oxygens from the SF residues, create a stack of K^+^ binding sites where dehydrated K^+^ binds to induce a conductive conformation of the SF. This increases intersubunit interactions and confers a higher stability to the channel against thermal denaturation. Indeed, the fit of dehydrated K^+^ to its binding sites is fundamental to define K^+^ selectivity, an important feature of these channels. Nonetheless, the SF conformation can be modified by different effector molecules. Such conformational plasticity opposes selectivity, as the SF departs from the “induced-fit” conformation required for K^+^ recognition. Here we studied the KirBac1.1 channel, a prokaryotic analog of inwardly rectifying K^+^ channels, confronted to permeant (K^+^) and non-permeant (Na^+^) cations. This channel is pH-dependent and transits from the open state at neutral pH to the closed state at acidic pH. KirBac1.1 has the orthodox TVGYG sequence at the SF and thus, its behavior should resemble that of K^+^-selective channels. However, we found that when at neutral pH, KirBac1.1 is only partly K^+^ selective and permeates this ion causing the characteristic “induced-fit” phenomenon in the SF conformation. However, it also conducts Na^+^ with a mechanism of ion passage reminiscent of Na^+^ channels, i.e., through a wide-open pore, without increasing intersubunit interactions within the tetrameric channel. Conversely, when at acidic pH, the channel completely loses selectivity and conducts both K^+^ and Na^+^ similarly, increasing intersubunit interactions through an apparent “induced-fit”-like mechanism for the two ions. These observations underline that KirBac1.1 SF is able to adopt different conformations leading to changes in selectivity and in the mechanism of ion passage.

## 1 Introduction

**FIGURE 1:**
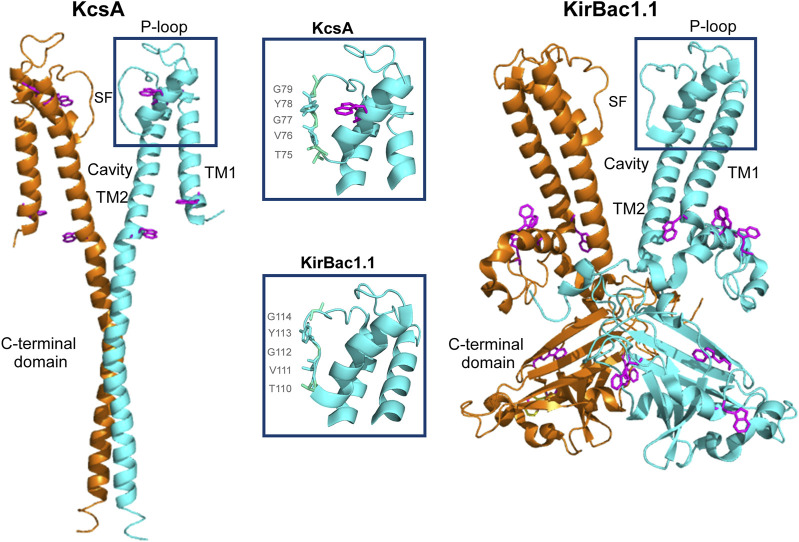
Structural representation of KcsA (PDB: 3EFF) and KirBac1.1 (PDB: 7SWJ) channels. For clarity, only two out of the four identical full-length subunits are shown for both channels. Each monomer consists of two transmembrane helices (TM1 and TM2) linked by the P-loop region and the selectivity filter (SF). Tryptophan residues are highlighted in magenta. The middle boxes provide a detailed view of the structure of the SF and nearby domains, showcasing the TVGYG sequence.

Potassium (K^+^) channels are a superfamily of complex membrane proteins widely distributed in different cell types of all living organisms. These channels contribute to the control of potassium flow, cell volume, release of hormones and neurotransmitters, resting potential, excitability and behavior (Jan and Jan, 1997), and their malfunction results in a number of important diseases termed channelopathies (Kumar et al., 2016; Demirbilek et al., 2019). In general, K^+^ channels are highly selective to allow permeation of K^+^ at near diffusion-limited rates, while passage of Na^+^, the biologically relevant competitor, is effectively prevented (Hille, 2001). The structural basis to explain such fundamental properties of K^+^ channels, selectivity and permeation, were initially provided by MacKinnon and co-workers by solving the high resolution structure of several prokaryotic potassium channels analogous to the eukaryotic counterparts. In particular, KcsA, a proton-activated prokaryotic channel from *Streptomyces lividans* (Schrempf et al., 1995), was the first of such structures to be solved (Doyle et al., 1998), revealing a homotetrameric protein in which, similarly to other K^+^ channels, the four subunits surround a central aqueous pore (Figure 1). Each KcsA subunit, comprises a short cytoplasmic N-terminal domain, two transmembrane segments, TM1 and TM2, and a long cytoplasmic C-terminal domain that contributes to form a tetrameric helical bundle which acts as one of the channel gates (inner gate). The TM1 and TM2 are connected by a pore region containing a tilted short-helix (pore helix), two loops and an ion Selectivity Filter (SF), which acts as a second channel gate (“outer” gate) and is believed to be the main responsible for ion selectivity. The SF, with the sequence TVGYG, unmistakably homologous to the more complex eukaryotic K^+^ channels, is located at the extracellular entry to the tetrameric channel pore region, where the backbone carbonyl oxygens from the SF sequence residues from all four subunits, point towards the pore axis to create a stack of multiple K^+^ binding sites at which the cation binds in a fully dehydrated form. Indeed, bound K^+^ have been seen, single file, in the crystal structure of KcsA and other K^+^ channels, and the perfect fit of the dehydrated K^+^ ions to the K^+^ binding sites within the SF, has been invoked as the most important factor to define K^+^ selectivity (MacKinnon, 2003; Zhou and MacKinnon, 2003). However, Na^+^ ions, being smaller, retain their hydration shell more strongly than K^+^, and therefore the energy required to dehydrate it is higher. In addition, this smaller size also hinders their interaction with the carbonyl oxygens that form the selectivity filter, which are at distances more in line with the larger size of the dehydrated K^+^, again contributing to impede the passage of Na^+^ along the filter (MacKinnon, 2003; Zhou and MacKinnon, 2003).

Nonetheless, the SF conformation can be modified by interaction with a number of effector molecules including specific membrane lipids, nearby channel protein domains or even by the permeating and/or blocking ions themselves in a concentration-dependent manner. This is to conclude that the SF exhibits conformational plasticity and it is believed that such plasticity opposes selectivity, as it causes the SF to depart from the “induced-fit” conformation required for K^+^ recognition (Renart et al., 2019, Renart et al., 2023; Giudici et al., 2022). The two gates of the KcsA channel are allosterically coupled, and ion conduction occurs when the “outer” gate permits ion passage (SF in a conductive conformation), simultaneously to the opening by acidic pH of the inner gate at the helical bundle (Cuello et al., 2017; Xu et al., 2017; Díaz-García et al., 2021).

Inwardly rectifying K^+^ (Kir) channels are a class of K^+^ channels which show an inward rectifying current-voltage relationship, due to a voltage-dependent block of the channel pore by intracellular polyamines and Mg^2+^ among other compounds (Hibino et al., 2010). Kir channels are ubiquitous, found in a diverse array of cell types, including cardiac muscle cells, neurons, blood cells, glial cells, epithelial cells, and oocytes. Their primary function is to influence resting membrane potential of cells, maintaining it close to the potassium equilibrium potential. This helps to reduce the likelihood of action potential firing in excitable cells, regulates potassium ion movement in non-excitable cells, and facilitates communication between the extracellular environment and the intracellular milieu. Thus, they are key elements in the physiology and pathophysiology of heart, the skeletal muscle, bones, blood vessels, kidneys, and neurons among others (Hibino et al., 2010; Cui et al., 2021).

As usual in ion channels, most of our knowledge on the structure of Kir channels comes from solving the crystallographic structure of prokaryotic analogs, such as the KirBac1.1 channel, used in this work, which has become a model channel for this subfamily of potassium channels (Kuo et al., 2003). This channel is also homotetrameric and each subunit has N-terminal and transmembrane regions fairly similar to those seen in KcsA. Moreover, and most relevant to our work, the two channels have an identical TVGYG sequence at their SF.

Previous studies on the structure of KirBac1.1 by crystallography have only captured a closed conformation of the channel. In this conformation, the SF showed a distorted structure with only three K^+^ binding sites. Although initially related to an active conformation (Kuo et al., 2003), other authors point to a non-active distorted structure. The open conformation has not been captured for this channel, but has been obtained for a mutant of another related prokaryotic channel, KirBac3.1. From this structure, if follows that the opening would occur at the TM2 bundle crossing in a manner similar to Kcsa. NMR studies support an allosteric communication between this inner gate and the external SF, acting as a second gate, also in a similar way to KcsA (Amani et al., 2020). The SF structure of the open conformation of KirBac1.1 has been studied through single-molecule fluorescence energy transfer, reporting a conformational change in this domain: when in K^+^, the SF fits the dehydrated ion becoming narrower, but in the presence of Na^+^, the SF becomes wider (Wang et al., 2019). This result points to a more flexible SF compared to KcsA. Another difference with this channel, is that of the C-terminal domain of KirBac1.1, much more complex than that of KcsA. It includes helical and β-structure segments and contains several subdomains, presumably involved in channel gating and modulation through a still not fully understood mechanism (Kuo et al., 2003; Wang et al., 2016) (Figure 1).

Related to the activity, patch clamp studies support KirBac1.1 as K^+^-selective, showing very complex currents with several subconductances, variability in gating kinetics, and no sign of inactivation (Cheng et al., 2009). Opposite to KcsA, KirBac1.1 is able to conduct Na^+^ in the absence of K^+^ (Wang et al., 2019). Interestingly, both channels are sensitive to pH, but in a completely opposite way. Thus, while acidic pH opens transiently the KcsA channel prior to inactivation, the KirBac1.1 channel, as well as most eukaryotic Kir’s, closes at acidic pH and remain open at neutral pH. The modulation of Kir channels through pH has significant physiological implications. In excitable cells like cardiac myocytes and neurons, changes in the pH, such as those during metabolic acidosis or alkalosis, could affect the activity of these channels, altering the resting membrane potential and action potential firing. Another example of the pH-Kir channel relationship is found in the regulation of potassium reabsorption in the distal nephron. Kir1.1 channels located there secrete excess K^+^ into the urine, playing a crucial role in K^+^ homeostasis. Interestingly, these channels are inhibited by H^+^, linking K^+^ transport and electrical activity to cellular H^+^ homeostasis. Inherited mutations in Kir1.1 that disrupt this pH-sensing mechanism can lead to hypersensitivity to H^+^, resulting in the hypokalaemic disorder type II Bartter syndrome (Rapedius et al., 2006).

Despite these previous findings, no systematic study of the structure of KirBac1.1 SF in the pH-induced open and closed states has been carried out, when confronted to classical permeant (K^+^) and non-permeant (Na^+^) cations. In this work we do so and also compare this behaviour with that previously observed in KcsA as a selective K^+^ channel of reference. As the SF sequence of the KcsA and KirBac1.1 channels are identical, a similar behavior in their interaction with the cations should in principle be expected. However, we found this not to be the case, suggesting that channel selectivity depends not only on the sequence, but also on the degree of conformational plasticity imposed on the SF as a major contributing factor.

## 2 Methods

### 2.1 KirBac1.1 heterologous expression and purification

A codon-optimized version of the I131C stable mutant (Wang et al., 2009) of KirBac1.1 was custom synthesized (GenScript Biotech, Rijswijk, Netherlands) to reduce its guanine-cytosine content and subcloned into pET-28a. Protein sequence is:

MNVDPFSPHS^10^SDSFAQAASP^20^ARKPPRGGRR^30^IWSGTREVIA^40^YGMPASVWRD^50^LYYWALKVSW^60^PVFFASLAAL^70^FVVNNTLFAL^80^LYQLGDAPIA^90^NQSPPGFVGA^100^FFFSVETLAT^110^VGYGDMHPQT^120^VYAHAIATLE^130^CFVGMSGIAL^140^STGLVFARFA^150^RPRAKIMFAR^160^HAIVRPFNGR^170^MTLMVRAANA^180^RQNVIAEARA^190^KMRLMRREHS^200^SEGYSLMKIH^210^DLKLVRNEHP^220^IFLLGWNMMH^230^VIDESSPLFG^240^ETPESLAEGR^250^AMLLVMIEGS^260^DETTAQVMQA^270^RHAWEHDDIR^280^WHHRYVDLMS^290^DVDGMTHIDY^300^TRFNDTEPVE^310^PPGAAPDAQA^320^FAAKPGEGDA^330^RPVLVPRGSR^340^SHHHHHH^347^

The C-terminal hexahistidine tagged KirBac1.1 channel was overexpressed in BL21 (DE3)-competent cells (Molina et al., 2004) with the following modifications. *E. coli* competent cells were transformed with the above construct following standard heat-shock procedures and plated overnight on LB agar. A single colony was picked up and grown overnight at 30°C in 100 mL of LB medium. This culture was diluted into 2 L of 2xYT medium and grown at 37°C to exponential phase (absorbance at 600 nm of ∼0.6). Protein expression was induced by 0.5 mM isopropyl β-D-thiogalactopyranoside at 30°C for 3 h. Cells were pelleted and suspended in 100 mL of buffer (20 mM HEPES, pH 7.5, 0.45 M sucrose) containing one EDTA-free protease inhibitor cocktail tablet (Roche, Madrid, Spain) and kept on ice for 1 h. The mixture was sonicated on an ice bath using a Branson probe-type apparatus and centrifuged for 45 min at 100,000 × g. Membrane proteins in this crude membrane pellet were solubilized in 40 mL of 20 mM HEPES, pH 7.5, 100 mM KCl, 50 mM imidazole and 10 mM n-dodecyl β-D-maltoside (DDM; Calbiochem, Madrid, Spain) for 2 h at room temperature. After centrifugation of insoluble remains (45 min at 100,000 × g), the supernatant was incubated with Ni^2+^- Sepharose 6 fast flow resin (Cytiva, Madrid, Spain) overnight at 4°C, placed into a column, and washed with 20 mM HEPES, pH 7.5, 100 mM KCl, 50 mM imidazole, and 5 mM DDM, until the absorbance at 280 nm was <0.01. The gel-bound protein was eluted using 15 mL of the previous buffer containing 500 mM imidazole. Protein concentration was routinely determined from the absorbance at 280 nm and always given in terms of KirBac1.1 monomers, using a molecular weight of 38.8 kDa and a molar extinction coefficient of 51,350 M^−1^cm^−1^ (Pace and Scholtz, 1997). The purified protein batches were checked by SDS-PAGE and BN-PAGE (Giudici et al., 2013).

### 2.2 Reconstitution of KirBac1.1

KirBac1.1 was reconstituted in asolectin liposomes for the activity assays, as previously reported (Poveda et al., 2019). The required amount of asolectin (Soybean, type II-S, Sigma, Madrid, Spain) was dissolved in chloroform:methanol (2:1, by volume) and the solvents removed using a rotary evaporator and vacuum. The dried lipid film was resuspended at 10 mg/mL in 10 mM HEPES, pH 7.0, 100 mM KCl and stored in liquid nitrogen. Before use, defrosted lipid suspensions were diluted to 5 mg/mL, then vortexed and extruded through 100 nm filters to obtain large unilamellar vesicles. DDM solubilized protein channels at approximately 1 mg/mL were added drop by drop to the lipid solution while being vortexed to give a lipid-to-protein ratio of 50:1 by weight. Bio-Beads SM-2 (Bio-Rad laboratories, Madrid, Spain) were added and the suspension was rocked overnight at 4°C to remove detergent. After discarding the Bio-Beads, the reconstituted liposome suspensions were collected by centrifugation for 65 min at 100,000 × g (TFT-70; Beckman, California, United States) and finally suspended in 10 mM HEPES, pH 7.0, 100 mM KCl. Samples were stored at −80°C (Giudici et al., 2022).

### 2.3 Electrophysiological recordings

For patch-clamp experiments, 3 µL of the above-reconstituted liposomes were placed on a clean glass slide and dried overnight in a desiccator chamber at 4°C and then rehydrated with 20 μL of 10 mM HEPES (pH 7.0), yielding multilamellar giant liposomes after a few hours of rehydration. A volume of 3–6 µL of the resulting giant liposome suspension were deposited onto a 3.5 cm Petri dish and mixed with 3 mL of buffer, the bath solution in the electrical recordings. Four different buffers were used either to fill the patch electrodes (pipette solution) or the bath solution, 10 mM MES pH 4 or 10 mM HEPES pH 7, containing either 200 mM of NaCl or KCl. Giga seals were formed on giant liposomes with borosilicate microelectrodes (Sutter Instruments, California, United States) of 3–5 MΩ open resistance. Standard inside-out patch-clamp recordings were performed using an EPC-9 (Heka Electronic, Lambrecht/Pfalzt, Germany) amplifier, at a gain of 50 mV/pA. Recordings were filtered at 1 kHz with an 8-pole Bessel filter. The holding potential was applied to the inside of the patch pipette, and the bath was maintained at virtual ground. An Ag−AgCl wire was used as the reference electrode. The data was analyzed with Clampfit 10.3 (Molecular Devices, Axon Instruments, San José, CA, United States). All measurements were made at room temperature (24°C). Reversal potentials were determined under bi-ionic conditions in order to obtain the permeability ratio of Na^+^ relative to K^+^, P_Na_^+^/P_K_^+^, from the Goldman–Hodgkin–Katz equation (Hille, 2001):

$$E_{rev}=\frac{RT}{F}\ln\left(\frac{P_{K^{+}}{\left[K^{+}\right]}_{0}+P_{{Na}^{+}}{\left[{\text{Na}}^{+}\right]}_{0}}{P_{K^{+}}{\left[K^{+}\right]}_{i}+P_{{Na}^{+}}{\left[{\text{Na}}^{+}\right]}_{i}}\right)$$ (1)

where *E*_*rev*_ is the calculated reversal potential (zero-current potential), *R* the gas constant, *T* the temperature (297 K), *F* the Faraday constant, [K^+^]_o_ and [K^+^]_i_ are the extracellular and intracellular K^+^ concentrations, respectively, and [Na^+^]_o_ and [Na^+^]_i_ refer to extracellular and intracellular Na^+^ concentrations, respectively.

Liquid junction potentials between the pipette and bath solutions were calculated by using Clampex 10.3 (Molecular Devices, Axon Instruments, San José, CA, United States) and routinely corrected. To measure the reversal potential of the channel currents under bi-ionic conditions, the holding potential was stepped to different voltages (10 mV steps), and the null current potential for each seal was the x-axis intersection point of the plotted I/V values.

### 2.4 Fluorescence-based activity assay

KirBac1.1 reconstituted in asolectin liposomes in 10 mM HEPES pH 7, either with 100 mM KCl or NaCl, was incubated for 15 min at room temperature with a stock solution of the fluorescent probe 9-Amino-6-chloro-2-methoxyacridine (ACMA) in methanol, at a final probe concentration of 100 µM. Samples were diluted 1:160 with N-methyl-d-glucamine (NMDG) buffer at different pHs in a 5 × 5 mm quartz cuvette to create a cation gradient. These NMDG buffers are composed of 10 mM HEPES for pH 7, and 10 mM Succinic acid for pHs 4, 4.5, 5, 5.5, 6, 6.5, all of them with 200 mM NMDG^+^ acting as a non-conductive cation. All experiments were conducted at 25°C using a PicoQuant F300 spectrofluorometer with excitation and emission wavelengths of 410 nm and 480 nm, respectively. Fluorescence reading was taken every second for about five minutes to obtain a stable baseline (*F*_*B*_) before the addition of carbonyl-cyanide m-chlorophenylhydrazone (CCCP) at a final concentration of 2 μM, prepared from a stock in DMSO, to initiate the ion flux. CCCP is a proton ionophore which enables the entry of protons to counter the efflux of ions conducted by KirBac1.1. This flux of protons is then monitored by the membrane-permeable pH-sensitive dye ACMA, which at neutral pH has bright fluorescence but is quenched upon protonation and is no longer membrane-permeable. Fluorescence was monitored for about 10–15 min, reading every second to get a stable fluorescence (*F*_*C*_) before the addition of valinomycin (or gramicidin), which provides a channel-independent path for K^+^ (or K^+^ and Na^+^) conduction. Both ionophores were used at a 10 nM concentration prepared from a stock solution in ethanol, to determine minimal fluorescence (*F*_*V*_). Normalized fluorescence was calculated using:

$$F_{N}=\left(F-F_{V}\right)/\left(F_{B}-F_{V}\right)$$ (2)

and the % relative K^+^ flux was calculated as:

$$\%flux=100x\left(F_{C}-F_{V}\right)/\left(F_{B}-F_{V}\right)$$ (3)

*%flux* vs. pH curves were fitted with a Hill equation after transforming pH into H^+^ concentration:

$$\%flux=F_{M}-\frac{F_{M}x{\left[H^{+}\right]}^{n}}{k^{n}{+\left[H^{+}\right]}^{n}}$$ (4)

where [H^+^] is the concentration of H^+^, *F*_*m*_ is the maximum flux, *n* is the Hill coefficient, and *k* is the H^+^ concentration at which half of the maximum flux is reached, from which *pH*_*0.5*_ is calculated (*pH*_*0.5*_ = -log(*k*)).

### 2.5 Fluorescence monitoring of cation binding through thermal denaturation

Thermal denaturation of KirBac1.1 channel was monitored as described previously (Renart et al., 2010) using a Varian Cary Eclipse spectrofluorometer to record the temperature dependence of the protein intrinsic emission fluorescence at 340 nm after excitation at 280 nm. In these experiments, the protein was diluted to 1 µM concentration in either 20 mM HEPES buffer, pH 7.0, containing 5 mM DDM or 10 mM Succinic acid buffer, pH 4.0, containing 5 mM DDM and always in the presence of a fixed amount of the opposite cation being tested. The starting stock is always 20 mM HEPES buffer, pH 7.0, 5 mM DDM and 100 mM KCl. In order to have the protein in Succinic acid buffer or NaCl, a previous dialysis is performed. For the cation titrations, aliquots from stock solutions of either NaCl or KCl were added to the samples prior to the thermal denaturation recordings to provide the desired final cation concentrations. The midpoint temperature of dissociation and unfolding of the tetramer (tm, in Celsius) was calculated from the thermal denaturation curves by fitting a two-state unfolding model to the data (Renart et al., 2024). The dissociation constants of the KirBac1.1-cation complexes (K_D_) can be estimated from:

$$\frac{\left|{∆T}_{m}\right|}{T_{m}}=\frac{\left|T_{m}-{\left(T_{m}\right)}_{0}\right|}{T_{m}}=\frac{R{\left(T_{m}\right)}_{0}}{{∆H}_{0}}\ln\left(1+\frac{\left[L\right]}{K_{D}}\right)$$ (5)

where *T*_*m*_ and (*T*_*m*_)_*0*_ refer to the denaturation temperature (in Kelvin) for the protein in the presence and absence of ligand, respectively, *R* is the gas constant, and *ΔH*_*0*_ is the enthalpy change upon protein denaturation in the absence of ligand.

### 2.6 Fluorescence spectral measurements

Fluorescence emission spectra were taken on a Varian Cary Eclipse spectrofluorometer using 0.5 cm path-length quartz cuvettes, as described in (Renart et al., 2006). The samples were excited at 280 nm, and the emission was recorded between 300 and 400 nm in 1 nm increments.

Changes in the fluorescence emission spectrum of KirBac1.1 where quantified from the intensity-weighted average emission wavelength < *λ* >:

$$<\lambda >=\frac{\sum_{i}\lambda_{i}I_{i}}{\sum_{i}I_{i}}$$ (6)

where *I*_*i*_ is the fluorescence intensity measured at a wavelength *λ*_*i*_.

< *λ* > vs. pH curves were fitted with a Hill equation as in the flux assay (Equation 4) but substituting ion flux by < *λ* >. This fitting was used again to calculate *pH*_*0.5*_.

Samples used in these experiments were prepared as in the thermal denaturation assays at a KirBac1.1 final concentration of 1 μM, in a buffer of 10 mM HEPES, pH 7.0, 150 mM KCl (or NaCl), 5 mM DDM, or 10 mM Succinic acid, (pH 4.0, 4.5, 5.0, 5.5, 6.0, 6.5), 150 mM KCl (or NaCl), 5 mM DDM. All measurements were made at room temperature (25°C).

## 3 Results

### 3.1 Functional studies

**FIGURE 2:**
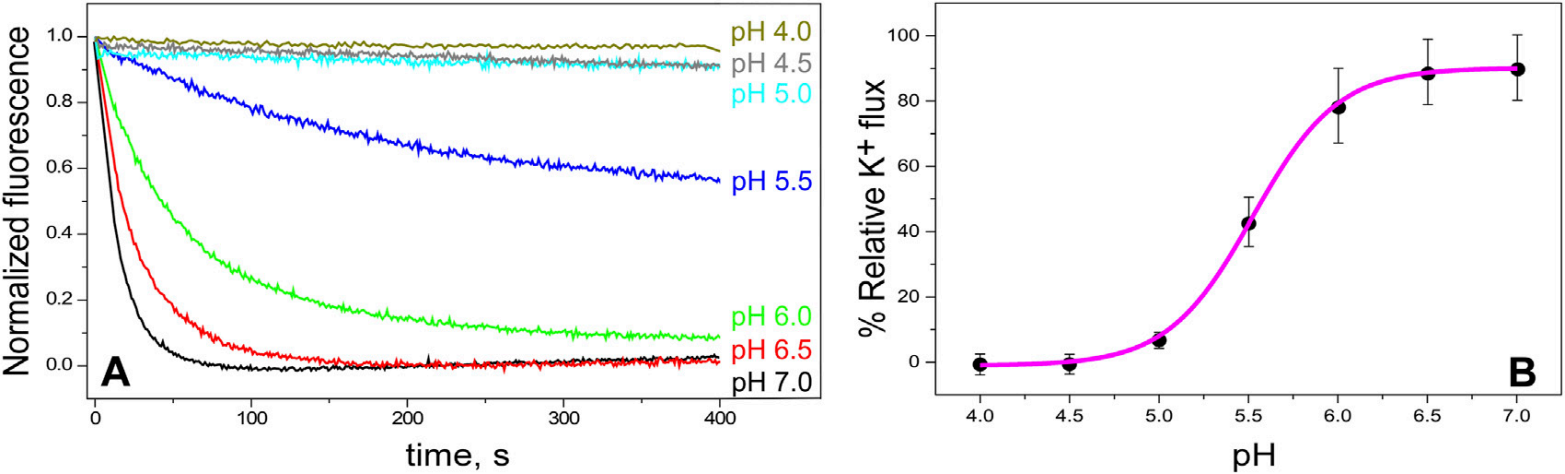
KirBac1.1 activity vs. pH. Panel **(A)**, representative time-dependent quenching curves of ACMA fluorescence after addition of the protonophore CCCP (zero time), indicative of K^+^ flux through the channel. The samples consist on KirBac1.1 reconstituted in asolectin liposomes (10 mM HEPES pH 7, KCl 100 mM intravesicular) at different extravesicular pHs (in 200 mM NMDG) (see Section 2). Panel **(B)** shows the relative K^+^ flux for KirBac1.1 at different pHs, calculated as described in Materials and Methods (Equations 2, 3), from curves such as those shown in Panel **(A)**. Several different batches of the purified channel protein were used to prepare the samples included in the panel. The results are the average flux (n = 3) ± S.D. The continuous line represents the fit of a Hill equation (Equation 4 in Section 2) to the data, from which *pH*_*0.5*_ is calculated.

Channel function in our purified KirBac1.1, reconstituted in asolectin lipid vesicles, has been assessed by two different procedures. First, we carried out a macroscopic assay of K^+^ flux by following fluorescence quenching (the ACMA/CCCP assay, as described in Methods). Figure 2A shows that, as expected for Kir channels, regardless of their prokaryotic or eukaryotic origin (Sepúlveda et al., 2015), channel opening is strongly pH-dependent. Figure 2B illustrates further such dependence, yielding an estimated pH_0.5_ for channel opening of 5.5 ± 0.1, which is similar to that reported from Rb^+^ fluxes in this same channel (Enkvetchakul et al., 2004). Overall, these experiments indicate that our purified KirBac1.1 channel is active and that, as an experimental system, the closed or the open channel states may be favored by selecting the adequate pH conditions.

**FIGURE 3:**
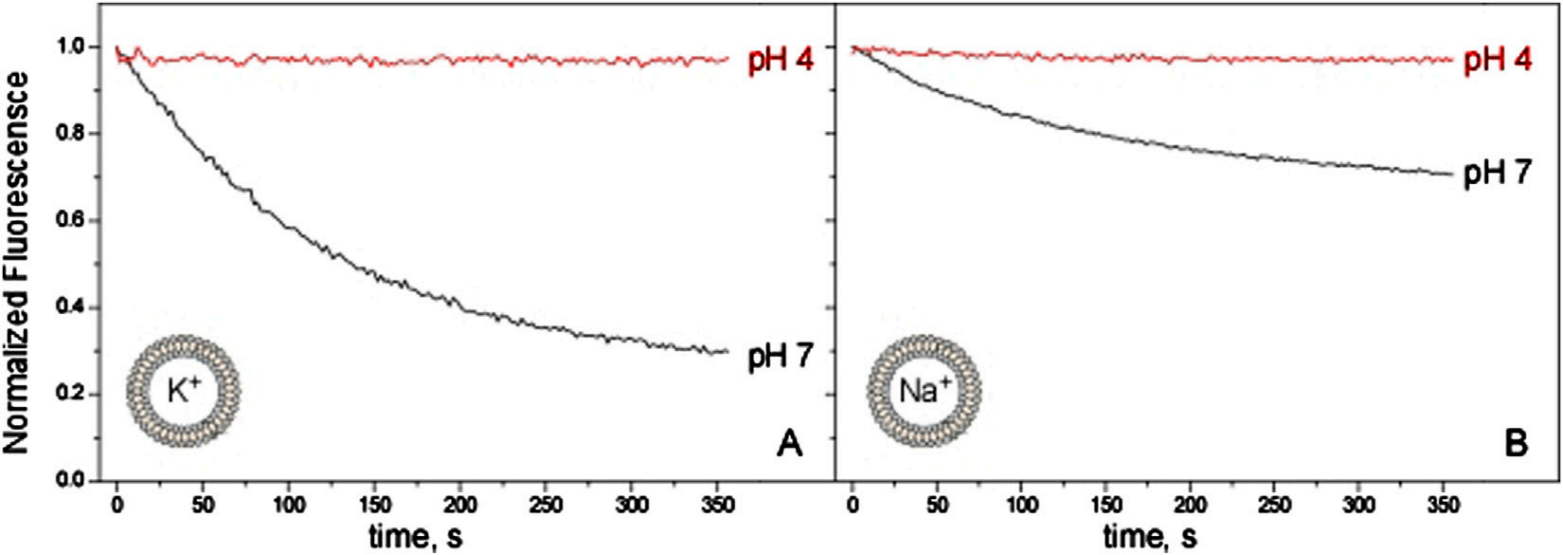
KirBac1.1 K^+^ and Na^+^ conduction at the macroscopic level. KirBac1.1 was reconstituted in asolectin liposomes (intravesicular buffers, 10 mM HEPES pH 7, KCl 100 mM, Panel **(A)**; 10 mM HEPES pH 7, NaCl 100 mM, Panel **(B)** and its activity was measured through the ACMA/CCCP assay. The same batch of purified protein was used to prepare all these samples to eliminate batch to batch variability and facilitate the comparison between the different experimental conditions. The extravesicular buffers are 10 mM HEPES pH 7, 200 mM NMDG^+^ (black) or 10 mM Succinic acid pH 4, 200 mM NMDG^+^ (red).

All the experiments reported in Figure 2 were conducted having K^+^ in the intravesicular compartment and a non-permeable cation, NMDG^+^, extravesicularly. Nonetheless, the same ACMA/CCCP assay can also be used to estimate cation selectivity by replacing K^+^ by Na^+^ inside the vesicles. In these conditions, if the KirBac1.1 channel were impermeable to Na^+^, no fluorescence quenching would be expected to occur. However, Figure 3 shows that Na^+^ efflux occurs at pH 7, although the process is less efficient than for K^+^. This suggests that KirBac1.1 is only partly selective for K^+^, although its SF is identical to that of KcsA, a channel markedly selective for K^+^ (LeMasurier et al., 2001; Montoya et al., 2017). Figure 3 also shows that at pH 4 no flux of neither K^+^ nor Na^+^ can be observed, indicating again that acidic pH favors the closed state of the channel, regardless of the ion used in the assays.

**FIGURE 4:**
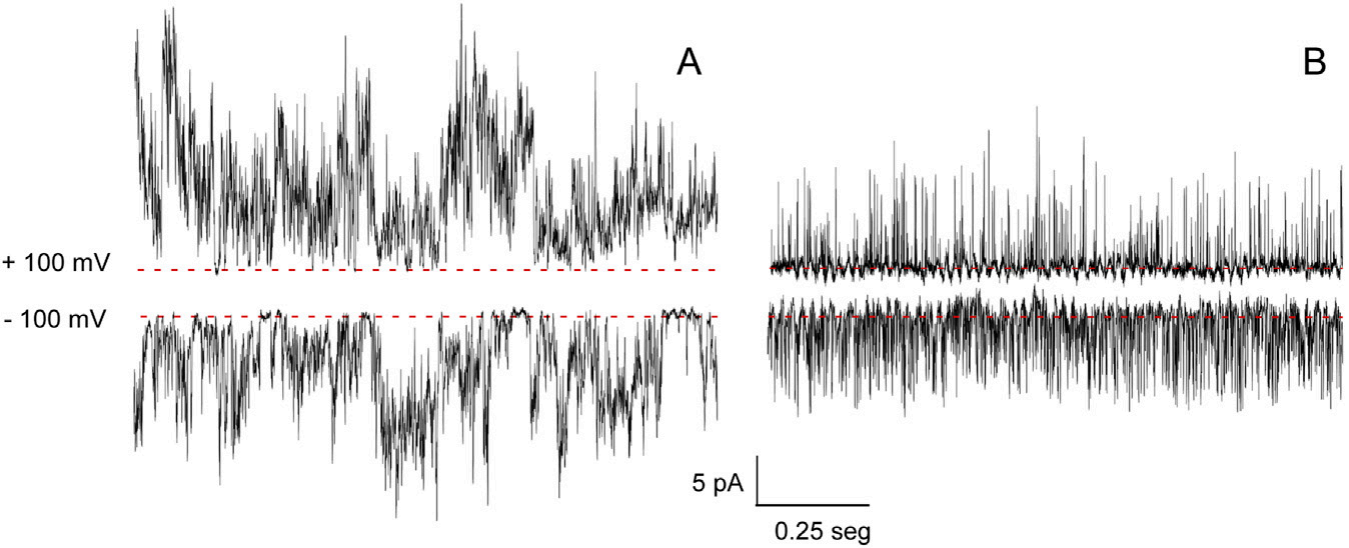
KirBac1.1 patch-clamp activity. Representative patch-clamp recordings at +100 (top) and −100 mV (bottom), under symmetric conditions of HEPES 10 mM, pH 7, KCl 200 mM **(A)** or HEPES 10 mM, pH 7, NaCl 200 mM **(B)**. The discontinuous line indicates the zero current level in each case.

To study the gating of KirBac1.1 in more detail, our second approach to measuring its function was to use patch-clamp methods on patches excised from reconstituted giant liposomes (Giudici et al., 2022). At pH 7 and 200 mM K^+^ at both sides of the patches (symmetrical conditions), an irregular activity with different current levels was observed (Figure 4A), which is similar to observations reported by other authors using reconstituted membranes with a different lipid composition (Cheng et al., 2009). Likewise, at pH 7 and under symmetrical 200 mM Na^+^ concentrations, channel activity is also observed (Figure 4B), although current levels were lower and the recordings even more flickery than those observed in K^+^.

**FIGURE 5:**
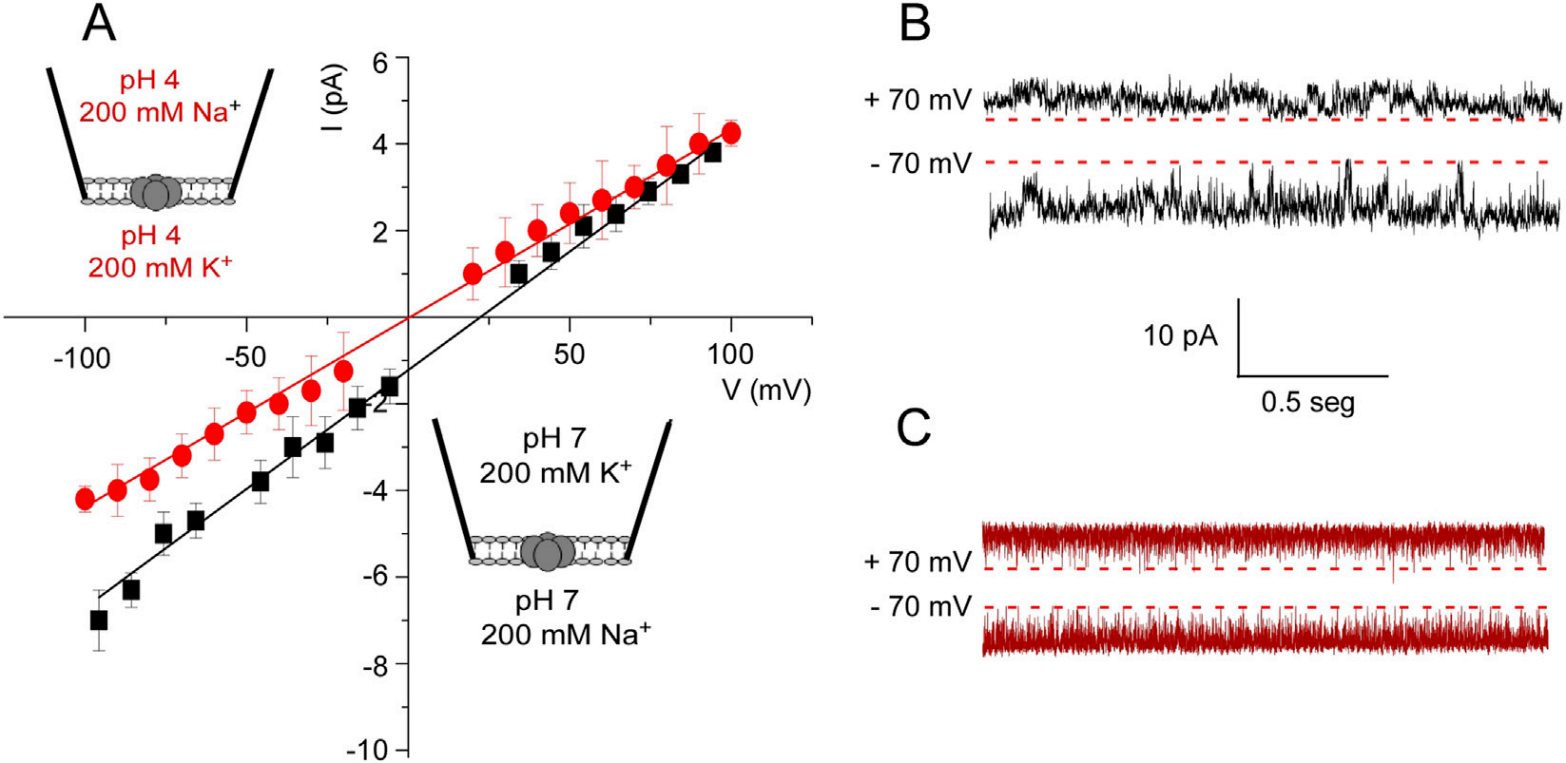
KirBac1.1 selectivity at pH 7 and pH 4. **(A)**, I-V curves are shown for the asymmetric ionic conditions included as insets within the Figure. The results are the average (*n* = 3) currents ±S.D. measured at different holding potentials. The continuous lines represent a linear fit to the data used to calculate the reversal potential. **(B, C)**, representative recordings at +70 and −70 mV for both asymmetric conditions are depicted. The discontinuous line indicates the zero current level in each case.

The relative selectivity of K^+^ versus Na^+^ was measured in membrane patches under asymmetric ionic conditions (200 mM K^+^ into the pipette solution and 200 mM Na^+^ in the bath). An intensity versus voltage (I/V) plot at pH 7 (Figure 5A) shows that outward K^+^ currents were slightly larger than Na^+^ inward currents. No signs of current rectification were observed because, as in many other Kir channels, rectification requires the presence of certain cofactors (Mg^2+^, spermidine and others) (Cheng et al., 2009) which are not present in our experimental system. In spite of the difficulties to measure current size in the recordings, a reversal potential of 21.5 mM was estimated (Equation 1), leading to a permeability ratio (P_K_^+^/P_Na_^+^) of 2.3. Such parameters are far from those estimated for truly selective K^+^ channels such as KcsA (LeMasurier et al., 2001; Montoya et al., 2017) and confirms the above observations from the ACMA/CCCP assays that the KirBac1.1 channel is only partly selective for K^+^.

At pH 4, most of the patches were silent, which is also in agreement with the ACMA/CCCP observations that the channels are inactive or closed at this pH. Nonetheless, channel electrical activity could still be observed in a percent of the patches under these conditions of acidic pH. Such activity is always fairly symmetric in terms of inward and outward current sizes and very flickery (Figures 5A, C). The I/V curves from such recordings yield a reversal potential of 0.8 mV and a relative permeability ratio near 1, indicating that at acidic pH, the channels that remain active are non-selective and able to conduct K^+^ or Na^+^ similarly.

### 3.2 Structural studies

The KirBac1.1 channel used in these studies contains a considerable number of tryptophan residues located at the cytoplasmic C-terminal protein domains (see Figure 1). Therefore, the channel is intrinsically fluorescent and we used such property to check spectroscopically whether there are detectable protein structural changes as a consequence of pH or in the presence of a given cation. All the following experiments were conducted with the purified protein in plain detergent (DDM) solutions. This eliminates both, the possible influence on the results caused by the presence of specific lipids (Singh et al., 2009; Amani et al., 2020) and excessive light scattering arising from the presence of lipid vesicles.

**FIGURE 6:**
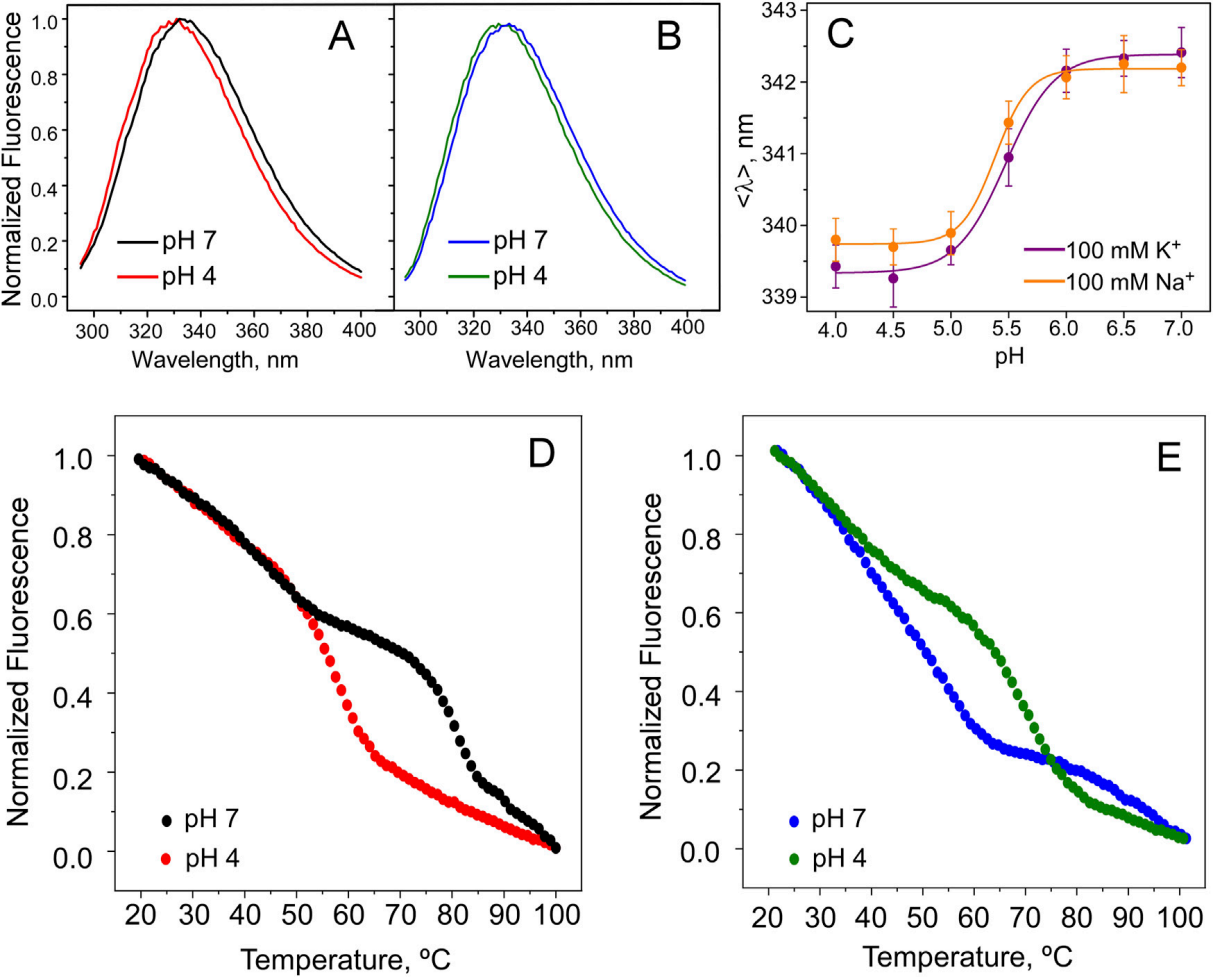
KirBac1.1 structure changes with pH. Normalized fluorescence emission spectra of KirBac1.1 at pH 7 and 4 (see Section 2), in buffers containing 100 mM KCl **(A)** or NaCl **(B)**. The effect of pH on the intensity-weighted average emission wavelength (<λ>, Equation 6) in buffers containing 100 mM KCl or NaCl **(C)**. The results are the average (*n* = 3) <λ> ± S.D., measured at different pHs. The continuous lines represent the fit of a Hill equation (Equation 4 in Section 2) to the data, from which *pH*_*0.5*_ is calculated. The channel thermal denaturation assay at pH 7 and 4 in buffers containing 100 mM KCl **(D)** or NaCl **(E)**. In all cases, purified KirBac1.1 solubilized in detergent (DDM) is used.

Figures 6A, B show that, regardless of whether the samples were prepared in a Na^+^ or K^+^ buffer, the fluorescence emission spectrum undergoes a small but reproducible blue shift when the pH swiches from pH 7 to 4. This suggests that pH causes structural changes in the cytoplasmic C-terminal region of the protein channel. The occurrence of conformational changes with pH was also suggested in previous FRET and NMR studies (Sadler et al., 2016; Amani et al., 2020). Interestingly, the fluorescence spectral shifts observed here, although small, allow to do pH titration experiments (Figure 6C), which clearly show a sigmoidal variation, very similar to that observed in the ACMA/CCCP assays of ion flux versus pH. The similarities between the pH_0.5_’s obtained from application of the two techniques, monitoring very different experimental observables (the spectral shift and the ion flow), suggest that changes in the channel structure accompany the functional changes observed in the transition from the closed to the open channel states. Figure 6C also shows that, despite the different effects of Na^+^ or K^+^ on the SF described below, the fluorescence spectral changes were similarly observed regardless of the presence of either Na^+^ or K^+^ in the samples. This is not too surprising because, as stated above, the tryptophane reporter groups in KirBac1.1 are located at intracellular protein domains, while cation binding takes place at the distant channel SF (Kuo et al., 2003). In contrast to these observations on KirBac1.1, previous studies using KcsA, in which the reporter trytophane groups are adjacent to the selectivity filter, demonstrated an exquisite sensitivity of the protein fluorescence to the binding and the filling of the cation binding sites at the SF (Zhou and MacKinnon, 2003; Renart et al., 2006, 2010; 2019).

From the KcsA and NaK channels, we also learned that K^+^ and other cation’s binding to the SF, bridge together the subunits within the tetrameric channel structure, conferring a large increase in the protein thermal stability and protecting it against tetramer dissociation and partial unfolding (Renart et al., 2010; Giudici et al., 2022). Thus, we decided to use the thermal denaturation assay to check whether cation binding causes also changes in the stability of the KirBac1.1 channel. Figure 6D shows that in 100 mM K^+^, the interaction of K^+^ with the channel is much stronger at pH 7, with the open channel state, than with the closed channel at pH 4, with T_m_ values (the mid-point temperature of protein denaturation) increasing considerably (up to 10°C–20°C for different preparations). In contrast, in 100 mM Na^+^ (Figure 6E), the interaction between Na^+^ and the protein channel seems stronger with the closed channel state at pH 4.

**FIGURE 7:**
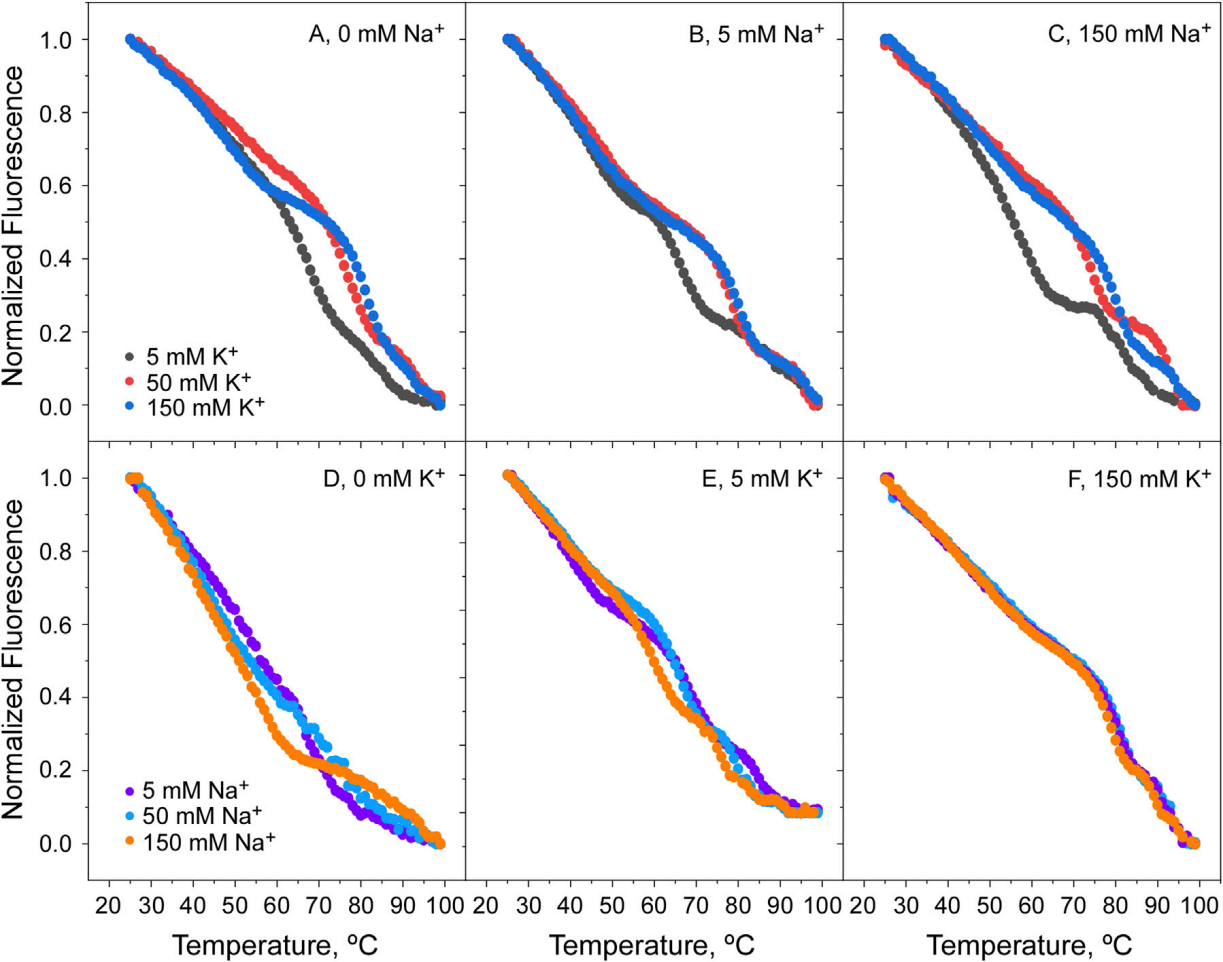
Thermal denaturation of KirBac1.1 at pH 7. Panels **(A–C)** show that raising the K^+^ concentration, the Tm of protein denaturation increases, indicative of its binding to the channel SF, either in the absence **(A)** or presence of NaCl at 5 and 150 mM (**(B, C)**, respectively). On the contrary, Panels **(D–F)** show that the increase in Na^+^ concentration has no effect on the Tm for protein denaturation, either in the absence **(D)** or the presence of KCl at 5 and 150 mM (**(E, F)**, respectively). In the experiments shown in this and in the following Figures, it should be noted that samples for K^+^ titrations contained an initial 5 mM Na^+^ as the starting point for the titration. Likewise, samples for Na^+^ titrations started with an initial 5 mM K^+^. This is to prevent protein aggregation due to the absence of cations in the solutions.

Since the results from Figures 6D, E indicate important differences in the binding of K^+^ and Na^+^ to the open and closed states of the channel, titrations experiments were conducted with the two cations competing with each other to further illustrate the binding processes taking place. At pH 7, when the channel is in the open state, the addition of K^+^ increases considerably the protein thermal stability, regardless of the presence of increasing concentrations of Na^+^ (Figures 7A–C). As extensively proven with KcsA and NaK channels (Renart et al., 2010, 2023; Giudici et al., 2022), this suggests that K^+^ entering into the KirBac1.1 selectivity filter favors the “induced-fit” conformation in which the channel’s subunits get closer together, bridged by the coordinating bound cations, thus, resulting in an increased thermal stability. On the contrary, such increased stability associated to the “induced-fit” phenomenon is not observed in the Na^+^ titrations (Figures 7D–F), in which the only stabilization observed comes from the presence of the fixed K^+^ concentrations used in the experiments. This leads to the conclusion that, despite being a permeable species through the KirBac1.1 channel, Na^+^ does not bind to the selectivity filter, or if it does, it is unable to cause the “induced-fit” conformation observed upon K^+^ binding.

**FIGURE 8:**
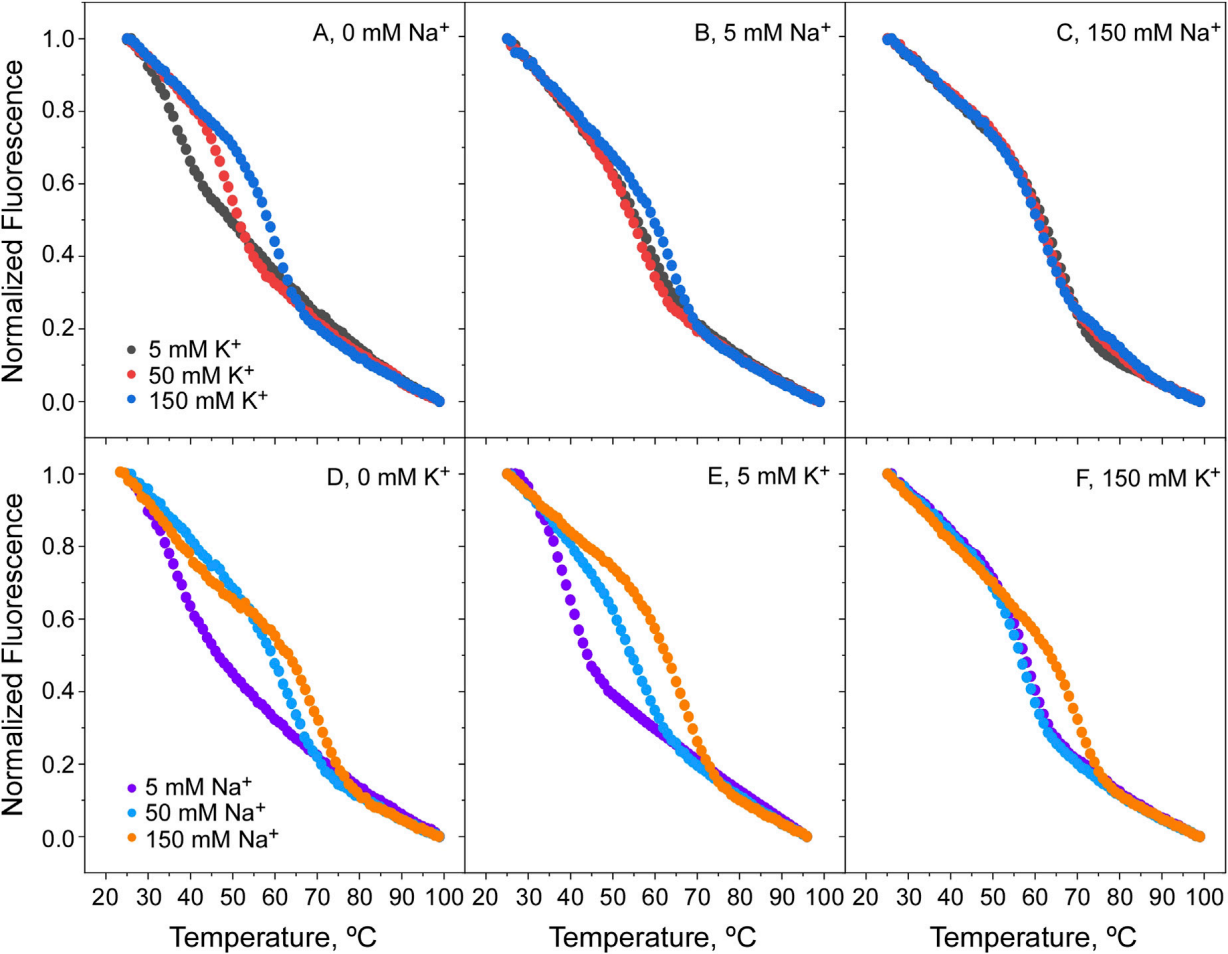
Thermal denaturation of KirBac1.1 at pH 4. Panels **(A)** shows that raising the K^+^ concentration, the protein denaturation Tm increases, indicative of its binding to the channel SF in the absence of Na^+^. However, as the concentrations of Na^+^ increase (5 and 150 mM, Panels **(B, C)**, respectively) the stabilizing effect of K^+^ is greatly diminished. Panel **(D)** shows that raising the Na^+^ concentration also increases the protein denaturation Tm in the absence of K^+^, indicative that at pH 4, Na^+^ binds to the channel SF. Furthermore, as the K^+^ concentration is increased (5 and 150 mM, panels **(E, F)**, respectively), the stabilizing effect of Na^+^ is still present although diminished.

Titration experiments similar to those above were conducted also at pH 4, where the channel is in a closed state, with markedly different results. Under this condition, K^+^ continues to bind and stabilize the channel in the absence of Na^+^, but once Na^+^ is added at higher concentrations, there is an additional stabilization caused by this ion (Figures 8A–C). This suggests that in the closed channel state, both cations compete with each other to bind to the channel and cause a phenomenon similar to the “induced-fit” in the selectivity filter conformation, leading to thermal stabilization. It is tempting to speculate that this similar ability of the two cations to act on the selectivity filter, could be related to the observed lack of cation selectivity observed in the functional studies described above. Likewise, in the Na^+^ titrations shown in Figures 8D–F, it is observed that Na^+^ strongly stabilizes the channel’s closed state, similarly to the stabilization caused by K^+^ on the open channel state from above. Moreover, in the presence of competing identical concentrations of Na^+^ and K^+^ (Figure 8F) it is observed that Na^+^ is even more effective than K^+^ in stabilizing the protein, suggesting that the affinity of Na^+^ for binding to the closed channel could be even higher than that for K^+^ at this acidic pH.

**FIGURE 9:**
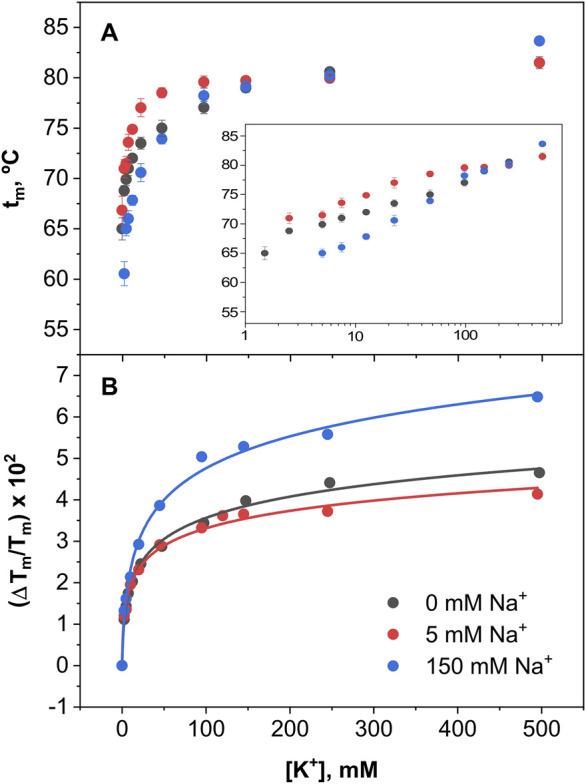
Binding of K^+^ to KirBac1.1 at pH 7. Panel **(A)** illustrates K^+^ binding to KirBac1.1 SF at three different fixed Na^+^ concentrations, monitored through the increase of the midpoint denaturation temperature. The results are the average (*n* = 3) tm (in Celsius) ± SD. Inset to panel **(A)** are semi-log plots to better illustrate the ion binding process. Panel **(B)** shows the fitting of the Equation 5 to the experimental data from Panel **(A)** (see Section 2), from which the apparent K_D_ values for the above binding events are calculated (Table 1).

In order to estimate the apparent dissociation constants for the above binding processes, Equation 5 as described in Methods, was fitted to the results from the titration experiments under competing conditions. Such fitting procedure has been used extensively in the past, mainly in studies on cation binding to the KcsA channel (Renart et al., 2017, 2024). Figure 9 shows the results from K^+^ titrations at pH 7, where the channel is in an open state. No results from Na^+^ titrations are included in the Figure because, as indicated above, the presence of Na^+^ does not result in any thermal stabilization of the channel under this condition. This Figure shows single exponential curves for K^+^ binding in the presence of three different concentrations of Na^+^, suggesting the existence of a single set of K^+^ binding sites, which are essentially unaffected by the simultaneous presence of Na^+^ and shows K^+^ dissociation binding constants in the millimolar range Table 1). Such values are similar to those low affinity constants exhibited by the KcsA channel for K^+^ binding, which were related to the conformation of the SF allowing K^+^ permeation (Renart et al., 2010).

**TABLE 1:** Apparent dissociation constants (K_D_’s) for the binding of K^+^ and Na^+^ to KirBac1.1 at pH 7 and 4 (see Section 2).

Binding K^+^							Binding Na^+^					
pH	0 mM Na^+^		5 mM Na^+^		150 mM Na^+^		0 mM K^+^		5 mM K^+^		150 mM K^+^	
	K_D_ (mM)	95% CI	K_D_ (mM)	95% CI	K_D_ (mM)	95% CI	K_D_ (mM)	95% CI	K_D_ (mM)	95% CI	K_D_ (mM)	95% CI
7	3.3	(2.8–3.9)	5.4	(5.2–5.6)	6.5	(5.1–8.2)	—	—	—	—	—	—
4	3.0 × 10^1^ ^a^	(2.2–4.1) × 10^1^	9.9 × 10^1^ ^a^	(0.6–1.6) × 10^2^	—	—	6.0	(4.3–8.3)	2.7 × 10^1^	(2.4–3.1) × 10^1^	1.5 × 10^2^	(0.9–2.5) × 10^2^

**FIGURE 10:**
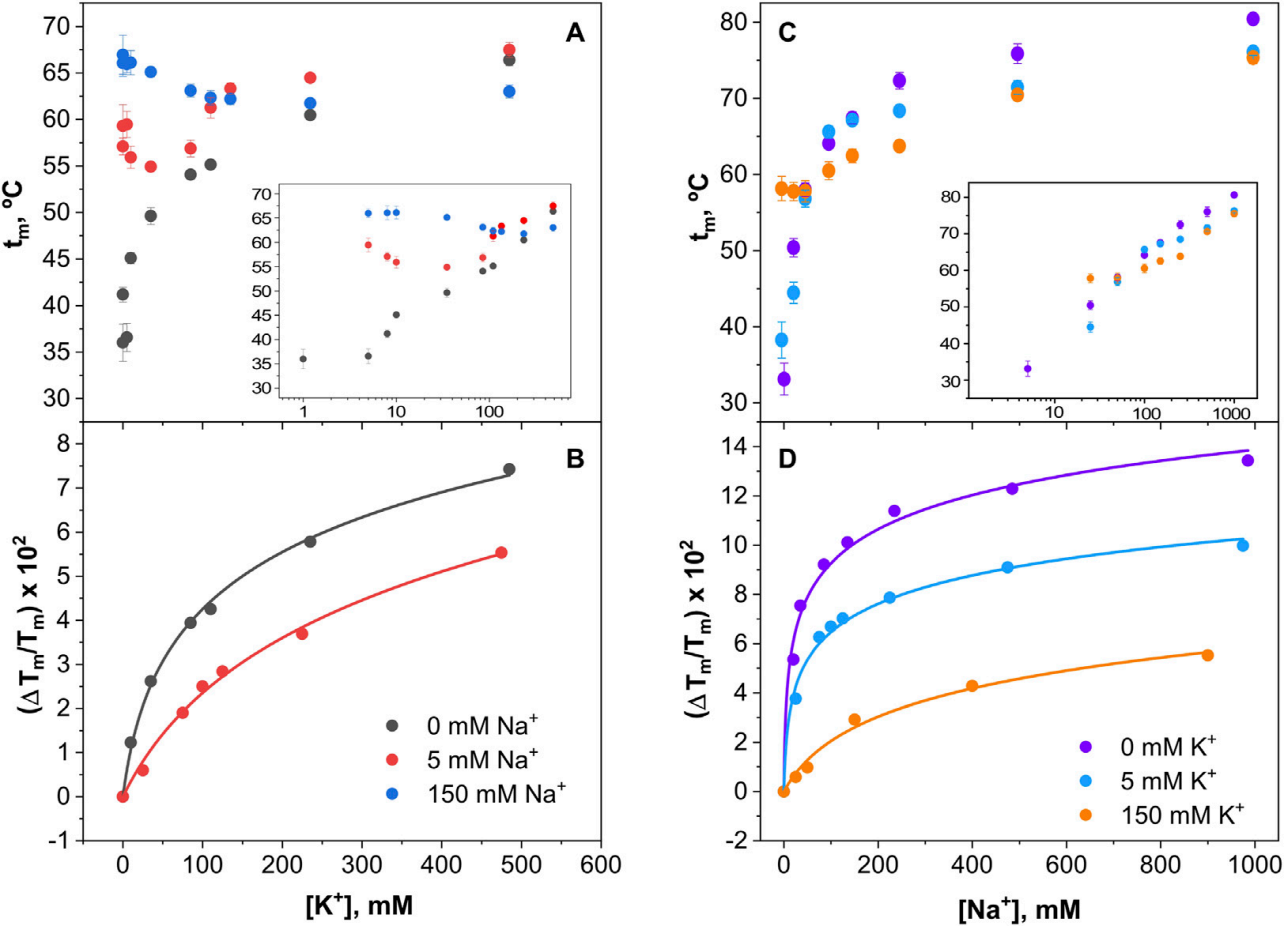
Binding of K^+^ and Na^+^ to KirBac1.1 at pH 4. Upper panels illustrate the K^+^ **(A)** or Na^+^ **(C)** binding to the KirBac1.1 SF at three different Na^+^ **(A)** or K^+^ **(C)** concentrations, monitored through the increase of the protein midpoint denaturation temperature. The results are the average (*n* = 3) tm (in Celsius) ± SD. Inset to panels **(A, B)** are semi-log plots to better illustrate the ion binding process. Panel **(B, D)** show the fitting of the experimental data from Panel **(A, C)**, respectively, to Equation 5 (see Section 2), from which the apparent K_D_ values for the above binding events are calculated (Table 1).

Mean values given here come from the experiments reported in Figures 9, 10. Since the estimated K_D_ values are derived from a logarithmic function (Equation 5) and since only parametric analysis is appropriate on the logarithmic scale for such data, we use the 95% confidence intervals of these values for statistical comparisons instead of giving mean ± SD values.

^a^ Significant difference with regard to the same sample at pH 7 (*p* < 0.05).

Figure 10 shows the results from the cation titration experiments at pH 4, where the channel is in a closed state. The binding curves are now more complex, except in the absence of cation competition, where the exponentials are nicely defined. Still, the analysis through Equation 5 allows an estimation of the dissociation constants for the binding of the cations to single sets of cation binding sites in the millimolar range or higher (Table 1), although the affinity to bind Na^+^ seems always higher than that for K^+^ binding. Such estimations clearly illustrate that, as a consequence of competition between Na^+^ and K^+^ to bind to the closed channel, their binding affinities decrease progressively as the concentration of the competing cation increases.

## 4 Discussion

Much of our current knowledge on the molecular mechanisms of key properties of ion channels, such as ion selectivity and permeability, derives from the high resolution structure of KcsA, a prokaryotic channel highly selective for K^+^ (Doyle et al., 1998; Zhou et al., 2001; Zhou and MacKinnon, 2004). In the earlier reports, the SF of such channel appeared as a stiff structure in which four ion binding sites, configured by the signature sequence TVGYG from the four channel subunits, accommodate dehydrated K^+^ in a very specific manner and excludes Na^+^. In spite of its stiffness, X-ray crystallography revealed two different, cation-dependent conformations of the SF: a conductive conformation in the presence of sufficient amounts of permeant cations and a non-conductive or collapsed structure when in low concentrations of permeant species or high concentrations of blocking species such as Na^+^, (Morais-Cabral et al., 2001; Zhou et al., 2001; Zhou and MacKinnon, 2003). Interestingly, the progressive entrance of K^+^ into the SF brings closer together the channel subunits by about 4 Å (Renart et al., 2019), reinforcing intersubunit interactions and resulting in a large increase in the protein stability against thermal denaturation (Renart et al., 2010, 2023; Giudici et al., 2022). In other words, the non-conductive to conductive conformational transition can be easily monitored by following the increase in thermal stability of the protein. Moreover, the SF conformation is also influenced by amino acid residues that lie behind the signature sequence, such as the well-known Glu71, Asp80 and Trp67 inactivation triad, which forms a hydrogen-bond network that modulates the KcsA conductive state by affecting the conformation of the K^+^ binding sites (Cuello et al., 2010). Additionally, other potential modulating interactions of the SF, including those that might arise from protein-bound lipids, are currently under investigation (Poveda et al., 2019; Renart et al., 2023). All such interactions, which vary among the K^+^ channels superfamily, appear as necessary elements to complement the proper signature sequence at the SF in fully defining the selectivity and permeation properties. As an example, different NaK-derived channels, which have an identical or very similar SF amino acid sequence compared to KcsA (Giudici et al., 2022), exhibits little or no selectivity for K^+^ because of different restricting interactions between the SF and its surroundings. This causes that these channels have a remarkable pore conformational flexibility, as they exhibit either a wide-open conformation of the SF in Na^+^ [as Na^+^ channels do (Ulmschneider et al., 2013; Naylor et al., 2016)] or a tight “induced-fit” conformation in K^+^. This enables the accommodation of different ionic species and thus, favors poor or no selectivity. Moreover, the NaK-derived channels lack a high affinity K^+^ binding component and do not collapse in Na^+^ and thus, NaK channels cannot properly select K^+^ over other cations or reject Na^+^ by collapsing, as K^+^-selective channels like KcsA do. As discussed below, most of these features are also present in the KirBac1.1 channel studied here.

In this manuscript we have studied the pH-dependent, open and closed states of KirBac1.1. This channel has a SF with an identical amino acid sequence to KcsA, but a markedly different behavior in its interaction with Na^+^ or K^+^. In the open state, at pH 7, both K^+^ and Na^+^ conduction can be detected, but still, the channel conducts K^+^ better than Na^+^ and therefore, it should be considered a partially selective K^+^ channel, as reported by others authors (Enkvetchakul et al., 2004; Cheng et al., 2009). Cation binding measurements using the thermal denaturation technique (Renart et al., 2010, 2023; Giudici et al., 2022) revealed that the presence of K^+^ causes a concentration-dependent, large thermal stabilization of the protein. This is consistent with our previous observations on KcsA that K^+^ causes an “induced-fit” conformation of the SF around the bound cations, which bridges closer together the channel subunits within the pore and reinforces intersubunit interactions. In marked contrast with the effects of K^+^, the presence of Na^+^ within a large concentration range does not result in any alteration of the protein thermal stability and therefore, the possibility of an “induced-fit” conformational phenomenon similar to that seen with K^+^, is excluded. Still, Na^+^ is a permeant species through the KirBac1.1 channel and we have to assume that its permeation occurs through a different conformation of the SF. Using a fluorescence resonance energy transfer (FRET) technique, Wang and coworkers (Wang et al., 2019) explained fairly similar observations proposing a “dilated” state of the SF caused by Na^+^, as opposed to the constrained, “induced-fit” conformation seen in K^+^. Indeed, this would be similar to that observed in Na^+^ channels (Ulmschneider et al., 2013; Naylor et al., 2016), in which the large, fully hydrated or hemi-hydrated Na^+^ goes through the channel SF in an unrestricted manner, without interactions causing anything comparable to the K^+^ “induced-fit” phenomenon (Ulmschneider et al., 2013; Naylor et al., 2016).

Experiments similar to the above were conducted at pH 4, where the channel is in the closed state. Despite this, a percentage of our membrane patches in the patch-clamp recordings still remained active under this condition and therefore, we tested for both, Na^+^ and K^+^ conduction. Flickering currents of similar size were observed for both Na^+^ and K^+^, but in contrast with the above observations, the channel now shows no selectivity for neither cation and therefore, the KirBac1.1 channel in the closed state must be considered a totally non-selective channel. In an attempt to address cation binding to the channel under this condition, thermal denaturation experiments were also conducted in the closed channel state, at pH 4. Most surprisingly, these experiments showed that both, K^+^ and Na^+^, caused now thermal stabilization of the channel protein in a concentration-dependent manner. Moreover, the two cations compete with each other to bind to the channel, with Na^+^ seemingly having an even higher affinity than K^+^. As mentioned above, there is plenty evidence to suggest that K^+^-induced thermal stabilization arises from a reinforced interaction of the four subunits within the channel pore. Such K^+^ “induced-fit” conformational change is believed to be a characteristic feature in all K^+^ channels. Here, the observation that Na^+^ produces thermal stabilization on the closed state of the KirBac1.1 channel, suggests the occurrence of a similar “induced-fit”-like conformational phenomenon caused by Na^+^ on the channel SF. A most unusual feature of such Na^+^ effect it that it seems exclusively related to the closed channel state, as such phenomenon has not been observed in the channel open state at pH 7. The affinity of ions to bind the channel SF has been related to their conduction and therefore, to their selectivity (Montoya et al., 2017; Renart et al., 2024). Thus, the similarity found in the binding of K^+^ and Na^+^ ions to the KirBac1.1 SF at low pH, suggests a similar conducting mechanism for both cations, that could explain the lack of selectivity observed under this condition. At the moment, we have no evidence to pinpoint to the involvement of any particular interacting residue or protein domain able to modify the SF with pH in such a way. Nonetheless, ion selectivity alterations caused by pH in some Na^+^ channels have been attributed to changes in the protonation of certain SF residues (Finol-Urdaneta et al., 2014). In KirBac1.1, there are histidine residues (H117 and H124) nearby the SF, which in the KcsA sequence correspond to the strategic residues Y82 (near the inactivation triad) and R89 (a non-annular lipid binding site), respectively. Therefore, checking the possibility that nearby residues capable of protonating/deprotonating with pH cause local effects on KirBac1.1 SF might be worth pursuing. However, such histidine residues are not conserved in many other Kir channels, which makes the hypothesis less likely. A perhaps more attractive possibility is that, rather than local effects, the alteration of the SF by pH comes from allosteric effects, similar to the allosteric coupling between the inner and outer gates in KcsA (Xu et al., 2017; Díaz-García et al., 2021). In this respect, Amani and coworkers (Amani et al., 2020), using NMR techniques, have proposed an allosteric communication along the channel pore from the inner gate to the SF, which varies depending upon whether the channel is open or closed. We report here shifts in the fluorescence spectra of the channel in response to pH, indicative of changes in the tertiary structure at the cytoplasmic C-terminal portion of the channel, which includes the inner gate, where the reporter tryptophan residues are located. These pH-dependent changes, along with the effects of pH on the ion binding to the SF commented above, seems compatible with such allosteric linkage hypothesis. Furthermore, patch-clamp experiments in oocytes lends also apparent support to an allosteric communication between the pH regulated inner gate and the SF conformation in the eukaryotic Kir1.1 channel (Dahlmann et al., 2004).

Theresults discussed here on the behavior of the open and closed states of the KirBac1.1 channel, further underlines that different restricting interactions on the SF leads not only to changes in ionic selectivity, but also to drastic changes on the molecular mechanisms of ion passage through the channel pore, which has not been observed previously. Unfortunately, at the moment, there is a lack of high-resolution structural information at acidic pH on this or similar channels, which would be needed to fully confirm our conclusions. Securing such information, however, is obviously beyond the scope of this manuscript. On the other hand, since Kirbac1.1, as most other channel belonging to the Kirbac family, are expected to be sensitive to modulation by certain lipids and other compounds involved in defining the rectification properties, this report may provide the basis to undertake future studies to see if such additional interactions change the behavior of the purified protein alone reported here.

## Data Availability

The raw data supporting the conclusions of this article will be made available by the authors, without undue reservation.
